# Burden, clinical presentation and risk factors of advanced HIV disease in pregnant Mozambican women

**DOI:** 10.1186/s12884-022-05090-3

**Published:** 2022-10-08

**Authors:** Tacilta Nhampossa, Raquel González, Arsenio Nhacolo, Laura Garcia-Otero, Llorenç Quintó, Maura Mazuze, Anete Mendes, Aina Casellas, Gizela Bambo, Aleny Couto, Esperança Sevene, Khátia Munguambe, Clara Menendez

**Affiliations:** 1 Centro de Investigação Em Saúde de Manhiça (CISM), Rua 12, Vila da Manhiça, PO Box 1929 Maputo, Mozambique; 2 Instituto Nacional de Saúde (INS), Ministério de Saúde, Maputo, Mozambique; 3 Barcelona Institute for Global Health (ISGlobal), Barcelona, Spain; 4 Consorcio de Investigación Biomédica en Red de Epidemiología Y Salud Pública (CIBERESP), Barcelona, Spain; 5 Ministério de Saúde, Maputo (MISAU), Maputo, Mozambique; 6 Universidade Eduardo Mondlane (UEM), Faculdade de Medicina, Maputo, Mozambique

**Keywords:** Advanced HIV disease, Risk factors; pregnancy outcomes, Mozambique

## Abstract

**Background:**

Information on the frequency and clinical features of advanced HIV disease (AHD) in pregnancy and its effects on maternal and perinatal outcomes is limited. The objective of this study was to describe the prevalence and clinical presentation of AHD in pregnancy, and to assess the impact of AHD in maternal and perinatal outcomes in Mozambican pregnant women.

**Methods:**

This is a prospective and retrospective cohort study including HIV-infected pregnant women who attended the antenatal care (ANC) clinic at the Manhiça District Hospital between 2015 and 2020. Women were followed up for 36 months. Levels of CD4 + cell count were determined to assess AHD immune-suppressive changes. Risk factors for AHD were analyzed and the immune-suppressive changes over time and the effect of AHD on pregnancy outcomes were assessed.

**Results:**

A total of 2458 HIV-infected pregnant women were enrolled. The prevalence of AHD at first ANC visit was 14.2% (349/2458). Among women with AHD at enrolment, 76.2% (260/341) were on antiretroviral therapy (ART). The proportion of women with AHD increased with age reaching 20.5% in those older than 35 years of age (*p* < 0.001). Tuberculosis was the only opportunistic infection diagnosed in women with AHD [4.9% (17/349)]. There was a trend for increased CD4 + cell count in women without AHD during the follow up period; however, in women with AHD the CD4 + cell count remained below 200 cells/mm^3^ (*p* < 0.001). Forty-two out of 2458 (1.7%) of the women were severely immunosuppressed (CD4 + cell count < 50 cells/mm3). No significant differences were detected between women with and without AHD in the frequency of maternal mortality, preterm birth, low birth weight and neonatal HIV infection.

**Conclusions:**

After more than two decades of roll out of ART in Mozambique, over 14% and nearly 2% of HIV-infected pregnant women present at first ANC clinic visit with AHD and severe immunosuppression, respectively. Prompt HIV diagnosis in women of childbearing age, effective linkage to HIV care with an optimal ART regimen and close monitoring after ART initiation may contribute to reduce this burden and improve maternal and child survival.

**Supplementary Information:**

The online version contains supplementary material available at 10.1186/s12884-022-05090-3.

## Background

Prevalence of HIV infection (13.2%) in Mozambique continues being one of the highest worldwide [1]. Women living with HIV outnumbers men living with HIV; in some areas up to 30% of pregnant women attending antenatal care (ANC) clinics are HIV-infected [2, 3] while the rate of vertical transmission remains high across the country (13.4%) [4]. Efforts have been mounted in the last years to control the HIV epidemic. Significantly, the implementation of the Option B + in 2013 that includes provision of lifelong antiretroviral therapy (ART) to all HIV-infected pregnant and lactating women [5–7]. As a result, the number of HIV-infected individuals receiving ART has increased (1.402.902 people by December 2020), and the proportion of patients with advanced HIV disease (AHD) has significantly decreased from 73% in 2004 to 37% in 2014 [4, 8].

Despite these positive achievements, the high frequency of AHD among infected individuals is an important public health issue due to its high burden on the health system and the society as a whole, likely jeopardizing the achievement of the UNAIDS 95–95-95 targets in Mozambique [9]. Patients with AHD are at high risk of death from opportunistic infections (mostly tuberculosis, severe bacterial infections, and cryptococcal meningitis) [10, 11] even if they are on ART, and this risk increases with the reduction in CD4 cell count [12].

Previous studies on the burden of AHD in Mozambique were conducted in the general population focusing on the frequency of AHD at ART initiation [13]. No published information exists in the country on the burden and clinical presentation of AHD in pregnant women at ANC enrolment, which is critical to guide care management in this particularly vulnerable population. HIV infection in pregnancy is a well-known risk factor for poor pregnancy outcomes [14, 15]. In the absence of ART, HIV-infected pregnant women have eight times higher risk of maternal mortality than HIV-uninfected women [16–18]. Additionally, there is limited information on the duration of the immune-suppressive status in the post-partum period in women with AHD [18–20], and even less data on the effects of AHD on pregnancy outcomes, in Mozambique and in other southern African countries where the burden of the HIV epidemic is also high.

The objective of this study was to describe, in a rural area of southern Mozambique, the proportion of pregnant women presenting with AHD at first ANC clinic visit, its clinical presentation, risk factors for AHD, the immune-suppressive changes over time and the effects of AHD on pregnancy outcomes.

## Methods

### Study area

The study was conducted in Manhiça District, located 80 km north of the capital Maputo. The *Centro de Investigação em Saúde de Manhiça* (CISM) runs a Demographic and Health Surveillance System (DHSS) since 1996 [21]. There are twenty-one health centers in the District, one rural hospital and one referral District hospital, Manhiça District Hospital (MDH). The area is endemic for malaria [22, 23]. The HIV prevalence in women attending the ANC clinics was estimated to be 29% in 2010 and 23% in 2021 (3, Nhampossa et al., unpublished).

### HIV control program in Mozambique

At first diagnosis, HIV-infected individuals are registered in the HIV National Program Registry and receive a unique numeric identifier (NID), which allows patient tracking throughout the continuum of care [24]. A blood sample for CD4 + cell count is collected at diagnosis and repeated only if therapeutic failure is suspected, while HIV viral load is measured six months after ART initiation [25]. First line ART in adults consisting of tenofovir (TDF) /lamivudine (3TC) /efavirenze (EFV) or zidovudine (AZT) /3TC /nevirapine (NVP) and second line consisting of AZT /3TC /lopinavir-ritonavir (LPV/r) or abacavir (ABC) /3TC /LPVr were administered until 2019, transitioning to TDF + 3TC + dolutegravir (DTG) since then [26, 27]. All pregnant women attending the first ANC clinic are offered HIV testing. If the HIV test result is negative, then HIV testing is repeated every three months until the end of breastfeeding. If the HIV test is positive, prevention of mother to child transmission (PMTCT) of HIV with antiretroviral (ARV) drugs is provided on a monthly basis throughout pregnancy and breastfeeding. Cotrimoxazole prophylaxis (CTXp) is also given during pregnancy to HIV-infected women and continued until cessation of breastfeeding, regardless of the CD4 + cell count. Furthermore, tuberculosis prophylaxis with isoniazid is administered to the mother if she has not received it before or during pregnancy. Case management continues at the integrated chronic disease clinic after the end of pregnancy. Monthly clinical follow up of children born to HIV-infected women starts at one month of age to assess HIV infection status and nutritional and psychomotor development, and it ends three months after weaning, when the last HIV PCR is done. HIV-exposed infants receive ARV (AZT plus NVP) prophylaxis from birth up to 12 weeks of age together with CTXp beginning at four weeks of age. If HIV infection is confirmed in the infant, ART is initiated and CTXp maintained.

### Study design

This is a prospective and retrospective cohort study nested in the International Epidemiological Databases to Evaluate AIDS in Southern Africa (IeDEA-SA Platform), including an observational cohort of HIV-exposed and infected children and their mothers who were registered at the MDH [28]. Clinical information for the period prior to 2013 was retrieved from the clinical files, while from 2013 onwards prospectively collected during the ANC visit. All HIV-infected pregnant women living in the Manhiça’s DHSS area, enrolled at the MDH-ANC clinic and who consented to be included in the study from January 2015 to March 2020 were included and followed up for 36 months and their children for a month. The CISM DHSS and the ePTS (MoH electronic HIV patient tracking systems) databases were used to retrieve information on participant’s vital status and their CD4 + cell count over time, respectively.

### Definitions

Advanced HIV disease was defined as a CD4 + cell count < 200 cells/mm^3^ and/or WHO clinical stage III-IV. Severely immunosuppression was defined as a CD4 + cell count < 50cells/mm^3^ [12]. The value of the CD4 count at each time point of interest (6, 12, 18, 24, and 36 months) was estimated with the closest CD4 count value within ± 3 months of the time point of interest.

A difference in CD4 + cell count of 25 cells/mm^3^ between two measurements performed at an interval of about six months was considered clinically significant [29, 30]. Less than expected improvement in the CD4 + cell count was defined when the difference between two measurements performed at an interval of about six months was < 25 cells/mm^3^. Maternal education level was stratified into two groups, namely, no formal education (no education or did not complete primary education) and some formal education (at least completed primary education). Maternal underweight was defined as a body mass index (BMI) lower than 18.5, calculated from the women’s weight in kilograms divided by the square of her height in meters (kg/m^2^) [31]; anemia was defined as a hemoglobin concentration lower than 11 g/dL [32]; and the late postpartum period up as six weeks postpartum [33]. Preterm birth was defined as a gestational age at birth less than 37 weeks and low birth weight as a weight less than 2500 g [34].

### Statistical analysis

Analyses were conducted using Stata® software (version 14.0) (StataCorp LP, College Station, TX, USA). A descriptive analysis was performed with frequencies and percentages. Differences in the distribution of socio-demographic, clinical variables, and pregnancy and perinatal outcomes between pregnant women with and without AHD were assessed with the Fisher’s exact test for categorical variables and Mann–Whitney U test for continuous variables. Description of immune-suppressive changes included variation in CD4 + cell count median over time, the calculation of the difference of CD4 + cell count between visits and frequencies of women with less than expected improvement in the CD4 + cell count over time. The Jonckheere-Terpstra statistical test was used to assess trend of CD4 count over time (months after 1st ANC visit). The difference of CD4 + cell count between the first antenatal visit and six-month visit, and between the first antenatal and 12-months visit for all the pregnant women (total and stratified by AHD and without AHD) was calculated and summarized with the mean and a 95% confidence interval. Logistic regression was performed to evaluate: a) the association between AHD proportion and visit year and b) the factors associated with the two main outcomes: AHD at the first ANC visit and “less than expected improvement in the CD4 + cell count” in the postpartum visit. The adjustment covariates included in logistic regression analysis were those previously described in the literature [29, 30, 35, 36], i.e., age group, gestational age, ART at first ANC visit, ART regimen, body mass index and gravidity for AHD at the first ANC visit, and age group, gestational age, time on ART, WHO clinical stage and AHD for “less than expected improvement in the CD4 cell count” in the postpartum visit. Death rates were calculated and compared using a time-to-event analysis (Kaplan–Meier plot) and the *p*-value from long-rank test used to compare the two survival curves). Due to the large amount, the missing values were imputed using the Multivariate Imputation by Chained Eqs. [37]. Logistic regression was used to impute binary variables and polytomous logistic regression was used for categorical variables with more than two categories. No numeric variable needed imputation. A two-sided *p*-value < 0.05 was considered to indicate statistical significance in all the study analysis.

### Ethics approval and consent to participate

This study was performed in accordance with the Declaration of Helsinki and the National Bioethics Committee of Mozambique approved the study protocol in 2011 (327/CNBS/11), followed by subsequent annual renewals until the year 2020. After informing the pregnant women about the study objectives and methods, women consented for their participation, and that of their children, by signing a written informed consent. One copy of the consent form was handled to the participant.

## Results

### Frequency and temporal trends of advanced HIV disease

**Fig. 1:**
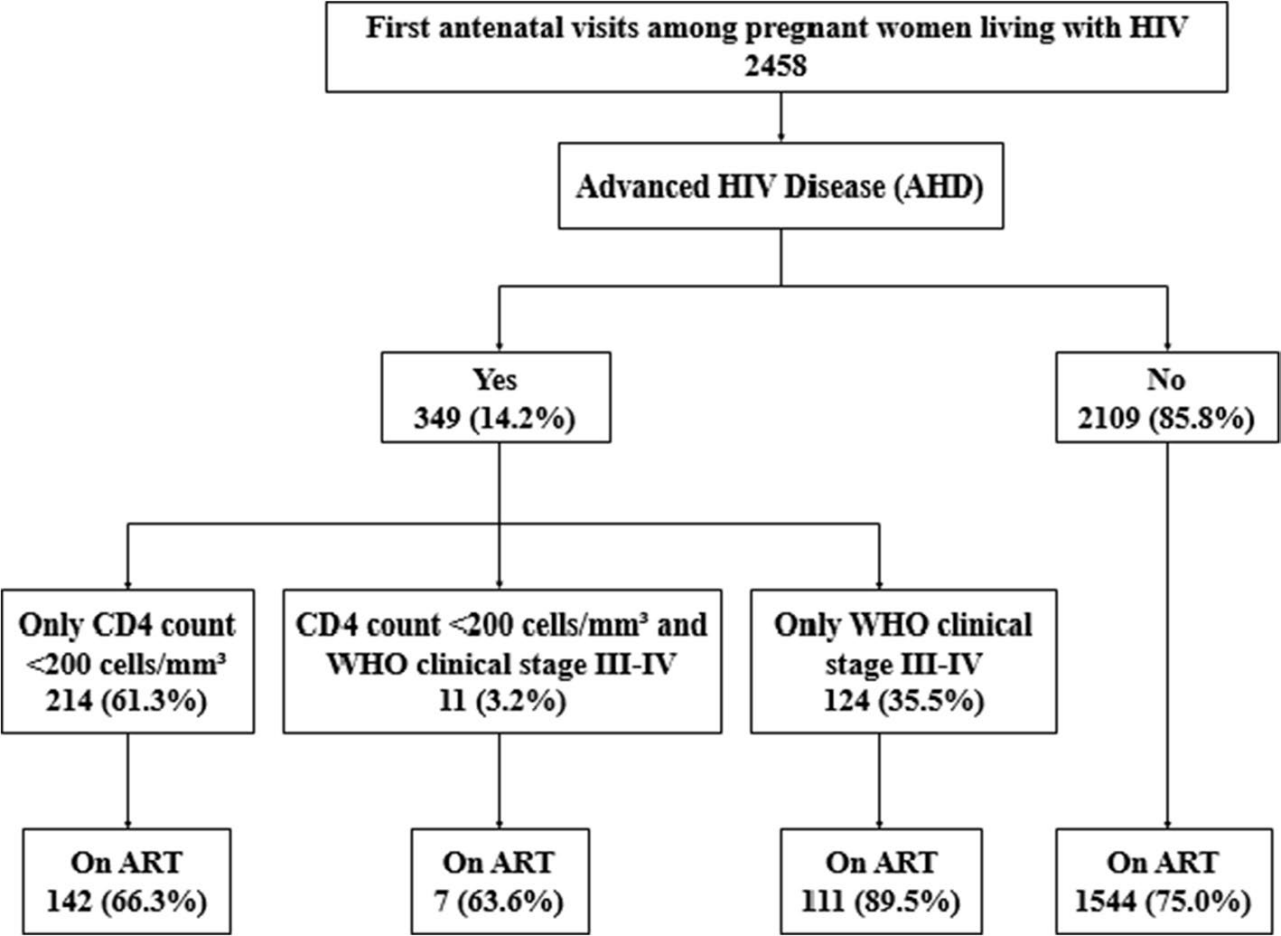
Study profile showing number and percentages of HIV pregnant women at first antenatal care with advanced HIV disease from 2015–2020 in Manhiça District Hospital

**Fig. 2:**
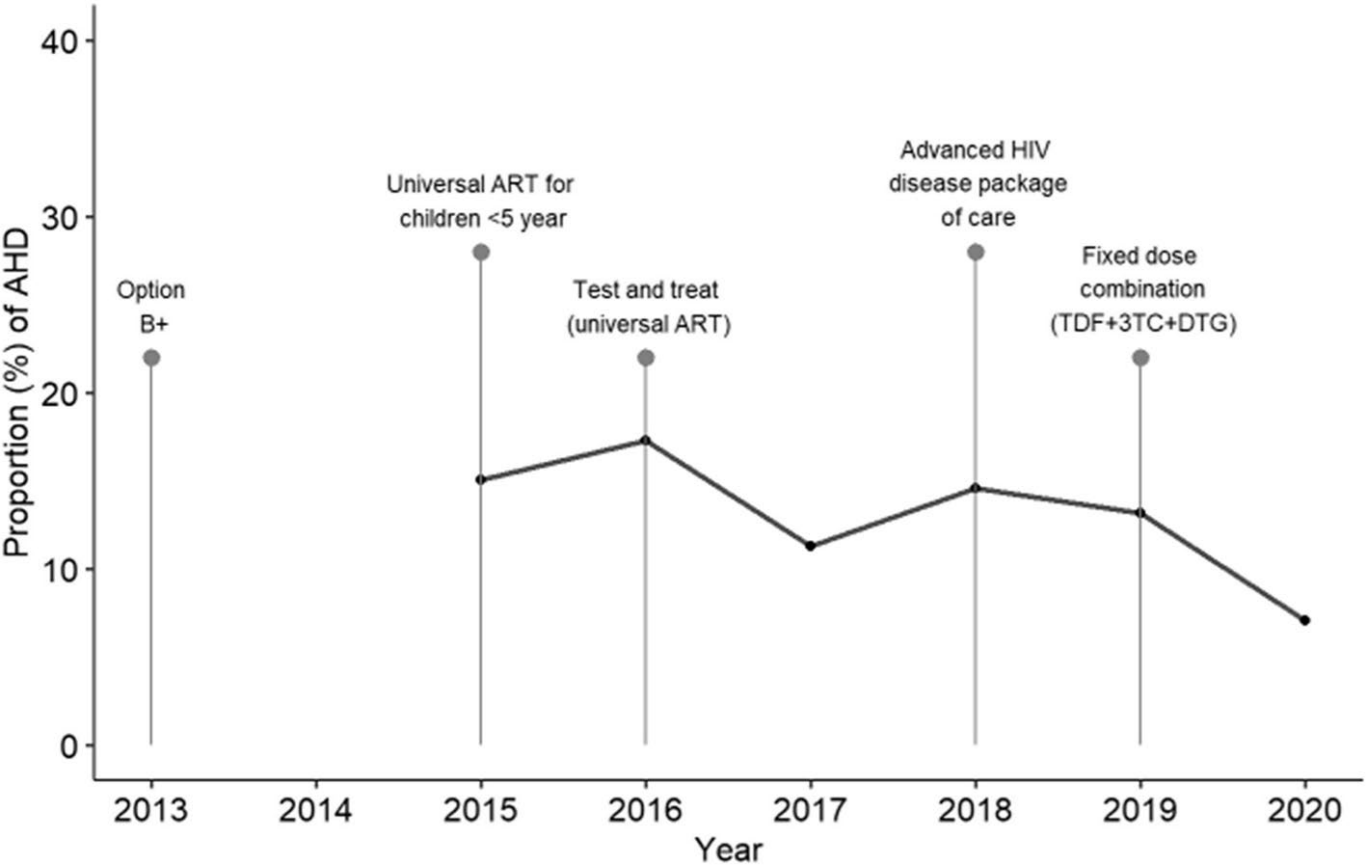
Proportion of pregnant women with advanced HIV disease per year at the Manhiça District Hospital and timing of implementation of HIV control interventions

Out of 2458 HIV-infected pregnant women who attended the first ANC clinic between 2015 and 2020, 349 (14.2%) presented with AHD (Fig. 1). Of them, 214 (61.3%) had a CD4 + cell count of < 200 cells/mm^3^, 124 (35.5%) were on WHO clinical stage III-IV, and 11 (3.2%) had both a CD4 + cell count of < 200 cells/mm^3^ plus WHO clinical stage III-IV. Forty-two (1.7%) women were severely immunosuppressed (< 50cells/mm^3^). No significant differences were detected in the annual proportion of women enrolling ANC with AHD over the five-year study period (*p* = 0.070) (Fig. 2).

### Characteristics of participants and risk factors for advanced HIV disease at first antenatal care clinic visit

Socio-demographic and clinical characteristics of enrolled women at first ANC clinic visit are shown in Table 1 according to AHD status. Pregnant women with AHD were significantly older than those without AHD (median age 30 *versus* 27 years, *p* < 0.001). The proportion of women with AHD increased with age, being 10.6% (25/236) for women aged < 20, 12.1% (73/601) for women aged 20–24, 13.9% (182/1314) for women aged 25–34 and 22.5% (69/307) for women older than35 years (*p* =  < 0.001). Median of CD4 + cell counts was 166 cells/mm^3^ (IQR: 97–477) and 548 cells/mm^3^ (IQR: 386–758) in women with and without AHD, respectively (*p* < 0.001). Women with AHD had been on ART for longer time than women without AHD (22.4 months *versus* 12.9; *p* < 0.001). Most [76.2% (32/42)] of severely immunosuppressed pregnant women were already on ART. Among women who were not on ART at first ANC visit, 14.5% (89/610) presented with AHD. The proportion of women receiving the AZT/3TC/NVP regimen was higher in those with AHD than in those without it [36 (10.9% (36/331) *versus* 3.6% (71/1991); *p* < 0.001]. The frequency of women on TB prophylaxis was significantly lower among those with AHD than in women without it [33.8% (118/349) *versus* 41.6% (878/2109); *p* = 0.006]. Tuberculosis was the only opportunistic infection diagnosed in women with AHD [4.9% (17/349)]. There were no differences in women’s educational level, marital status and gestational age by AHD diagnosis.

**Table 1:** Characteristics of pregnant women at first antenatal care visit by advanced HIV disease status

Variable		Advanced HIV disease		*P*-value
		Yes *N* = 349	No *N* = 2109	
Age (years): median (IQR)		30 (24–34)	27 (23–32)	< 0.001
Age group (years)	< 20	25 (7.2)	211 (10.0)	< 0.001
	20–24	73 (20.9)	528 (25.0)	
	25–34	182 (52.1)	1132 (53.7)	
	≥ 35	69 (19.8)	238 (11.3)	
Education	No formal education	4 (1.3)	22 (1.2)	0.776
	Some formal education	293 (98.7)	1721 (98.8)	
Marital status	Single	146 (46.1)	944(51.1)	0.080
	Married	13 (4.1)	34 (1.8)	
	Facto union	148 (46.7)	810 (43.9)	
	Widow	2 (3.2)	58 (3.1)	
Gestational age (weeks): median (IQR)		21 (16–24)	21 (17–25)	0.124
Gravidity	Primigravidae	48 (17.4)	222 (12.9)	0.047
	Multigravidae	228 (82.6)	1494 (87.1)	
Children alive: median (IQR)		2 (1–3)	2 (1–3)	0.040
WHO stage	I-II	214 (61.3)	2109	< 0.001
	III-IV	135 (38.7)	0	
CD4 + count (cells/mm^3^): median (IQR)		166 (97–477)	548 (386–758)	< 0.001
Women on ART at first ANC visit		260 (76.2)	1581 (75.0)	0.637
Time on ART (months): median (IQR)		22.4 (0–54.0)	12.9 (0–37.3)	< 0.001
ART regimen	TDF/3TC/EFV	282 (85.2)	1873 (94.1)	< 0.001
	AZT/3TC/EFV	2 (0.6)	1 (0.1)	
	AZT/3TC/NVP	36 (10.9)	71 (3.6)	
	AZT/3TC or *ABC*/3TC + LPV/r	11 (3.3)	46 (2.3)	
On cotrimoxazole prophylaxis		214 (61.3)	1384 (65.6)	0.130
On tuberculosis prophylaxis		118 (33.8)	878 (41.6)	0.006
Body mass index: median (IQR)		23.7 (22.0–26.1)	24.2 (22.3–26.7)	0.036
Underweight		7 (2.0)	34 (1.6)	0.650
Haemoglobin (g/dl): median (IQR)		10.5 (9.3)	10.6 (9.4–1.5)	0.703
Anemia		204 (58.5)	1212 (57.5)	0.770
Syphilis (RPR)		13 (4.3)	65 (3.8)	0.630

*P*-value for categorical variables is from Fisher exact test and for continuous variables is from Mann–Whitney U test. *IQR* Interquartile range, *ART* Antiretroviral therapy, *ANC* Antenatal care, *TDF* Tenofovir, *3TC* Lamivudine, *EFV*, Efavirenz, *AZT* Zidovudine, *NVP* Nevirapine, *RPR* Rapid plasma reagin. Anemia: hemoglobin concentration lower than 11 g/dL.

Independent risk factors for presenting AHD at first ANC clinic are shown in Table 2. After adjusting for covariates, the odds of AHD increased with age compared to women aged < 20 (*p* = 0.029) (aOR = 1.35, 95% CI: 0.66; 2.76 in women aged 20–24), (aOR = 1.87, 95% CI: 0.97; 3.60 in women aged 25–34) and (aOR = 2.52; 95% CI: 1.21; 5.25 in women aged > 35 years). The odds of AHD were higher among women receiving the AZT/3TC/NVP regimen (aOR = 3.29, 95% CI: 1.91; 5.67) and AZT/ 3TC / LPV/r or ABC regimen (aOR = 8.59, 95% CI: 2.56; 28.85; *p* =  < 0.001) compared to women on the TDF/3TC/EFV regimen. The Multivariate Imputation by Chained Equations sensitivity analyses did not find substantial differences, suggesting no major impact of missing data on the results (Tables 2b-Supplementary Information).

**Table 2:** Independent risk factors for advanced HIV disease

Variable			Unadjusted			Adjusted		
			OR	95%CI	*P*-value	OR	95% CI	*P*-value
Age group		< 20	1		0.013	1		0.029
		20–24	1.37	0.67; 2.77		1.35	0.66; 2.76	
		25–34	1.89	0.99; 3.61		1.87	0.97; 3.60	
		> 35	2.61	1.28; 5.33		2.52	1.21; 5.24	
Gestational age at the first ANC visit			0.98	0.96; 1.00	0.115	0.98	0.96; 1.01	0.146
ART start	Before first ANC visit		1		0.457	1		0.180
	After first ANC visit		1.15	0.80; 1.64		1.29	0.89; 1.87	
ART Regime	TDF/3TC/EFV		1		< 0.001	1		< 0.001
	AZT/3TC/NVP		3.53	2.07; 6.00		3.29	1.91; 5.67	
	AZT/3TC/LPV/r or ABC		9.05	2.73; 29.97		8.59	2.56; 28.85	
Body mass index			0.97	0.93; 1.01	0.134	0.96	0.92; 1.00	0.067

*P* value (LR-test). Number of observations: 1506 (unadjusted and adjusted ORs were based only on observations with complete information on all variables). *ART* Antiretroviral therapy, *ANC* Antenatal care, *TDF/3TC/EFV* Tenofovir/lamivudine/ efavirenze, *AZT/3TC/NVP Z*idovudine/ lamivudine/nevirapine

### Immunological changes and risk factors for CD4 + cell count reduction

**Fig. 3:**
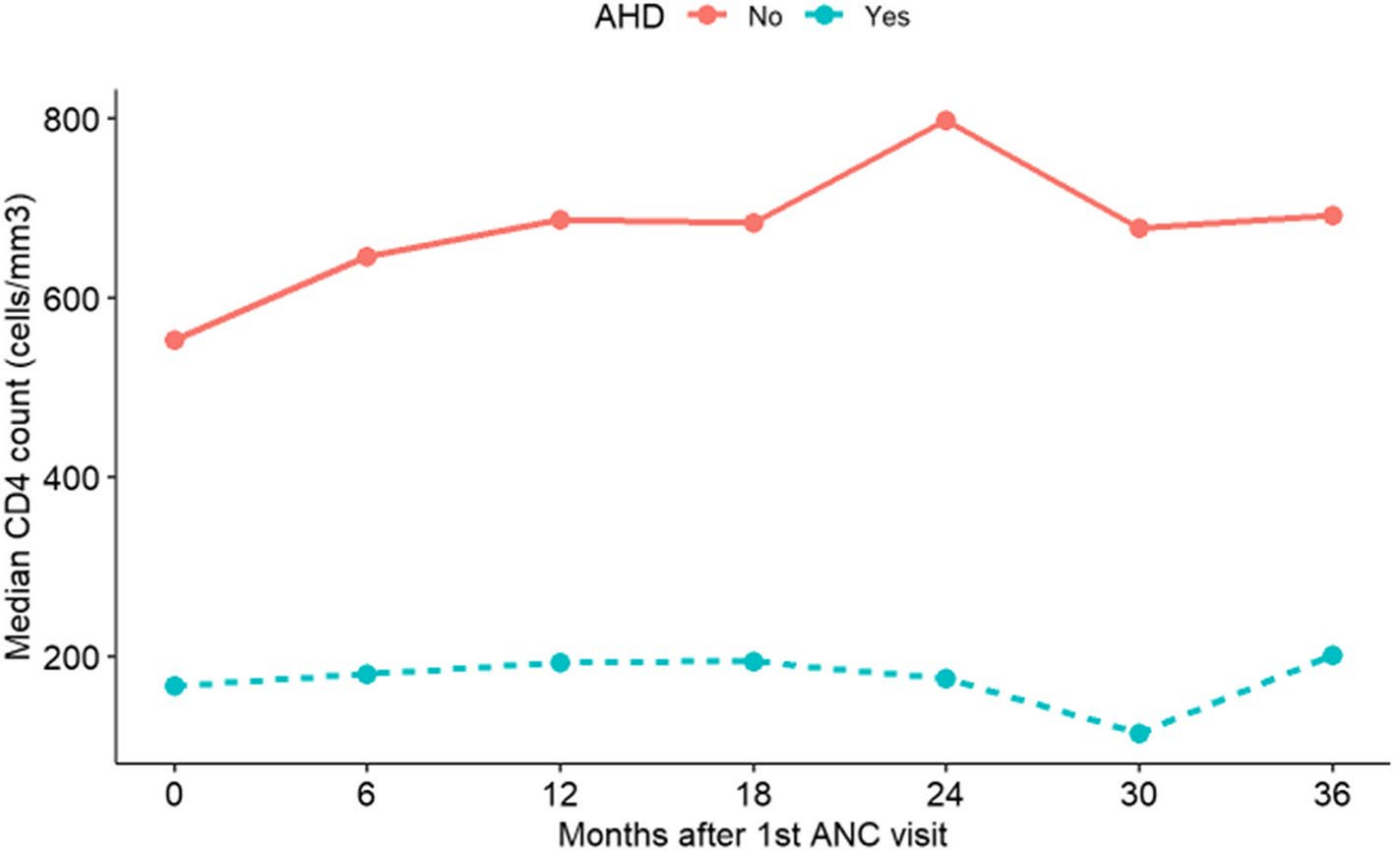
Median CD4 cell count over time in pregnant women with and without advanced HIV disease (AHD)

Figure 3 presents the median CD4 + cell count over time in pregnant women with and without AHD. We observed an increasing trend of the median CD4 + cell count in women without AHD during the 36 months follow-up (*p* < 0.001), however, this remained constantly below 200 cells/mm^3^ in women presenting with AHD at first ANC clinic visit.

In 12.5% (161/1288) of women (with and without AHD) at the six months visit and in 14.2% (148/1038) of them at the 12 months visit, there was a “less than expected improvement in CD4 + cell counts”. The reduction in CD4 + cell counts was of 274.5 cells/mm^3^ (95% CI: -323.76, -225.2) and 117.7 cells/mm^3^ (95% CI: -180.8, -54.5), at the six- and 12-months visits, respectively (Table 3). Table 4 shows the proportion of women with a “less than expected improvement in the CD4 + cell count” over time. Although not statistically significant, increases in the CD4 cell count at the 12- and 18-month visits (both coinciding with the breastfeeding period) were less common in women with AHD compared to those without it.

**Table 3:** CD4 + cell count difference between first antenatal care visit and subsequent visits by Advanced HIV disease status

Period	Difference of CD4 + count (cells/mm^3^) among all participants^a^			
	Advanced HIV disease			*P*-value
	Yes	No	All	
Between first ANC visit and 6 months visit (*n* = 1288)	94.3 (95% CI: 60.2; 128.4)	110.9 (95% CI: 82.3; 139.5)	108.7 (95% CI: 83.6; 133.9)	0.463
Between first ANC visit and 12 months visit (*n* = 1038)	136.0 (95% CI: 95.0; 177.9)	124.0 (95% CI: 102.9; 145.8)	126.0 (95% CI: 106.6; 145.4)	0.611
Period	Difference of CD4 + count (cells/mm^3^) among women with less than expected improvement in the CD4 + count *			
	Advanced HIV disease			*P*-value
	Yes	No	All	
Between first ANC visit and 6 months visit (*n* = 161)	-200.5 (95%CI: -285.1;-115.8)	-285.4 (95% CI: − 340.6; -230.2)	-274.5 (95% CI: -323.76; -225.2)	0.093
Between first ANC visit and 12 months visit (*n* = 148)	-118.3 (95% CI: − 215.1; -21.5)	-117.6 (95% CI: − 189.5; -45.6)	-117.7 (95% CI: − 180.8; -54.5)	0.990

*ANC* Antenatal care. ^a^Mean. Difference of CD4 cell count between visits was performed as follows: firstly, the absolute value of the difference of CD4 cell count between the first antenatal visit and six-months visit, and between the first antenatal and 12-months visit was calculated among all the pregnant women (total and stratified by AHD and without AHD). Afterwards, the same calculations were repeated only for women with negative differences (decrease in CD4) between visits

**Table 4:** Proportion of pregnant women with less than expected improvement in the CD4 + cell count by Advanced HIV disease status

Sample collection period after first ANC visit	Advanced HIV disease		Without Advanced HIV disease		*P* value
	N	Less than expected improvement in the CD4 cell count n (%)	N	Less than expected improvement in the CD4 cell count n (%)	
6 months^a^	100	12 (12.0)	1188	149 (12.5)	1.00
12 months^b^	83	16 (19.3)	955	132 (13.8)	0.230
18 months^c^	44	8 (18.2)	468	75 (16.0)	0.875

*AHD* Advanced HIV disease, *ANC* Antenatal care, *N* Number of available, *CD4* Count records in each period. Less than expected improvement in the CD4 cell count was considered if the difference between the two measurements performed at an interval of about six months was < 25 cells/mm^3^. ^a^Coinciding with childbirth or immediate postpartum; ^b^coinciding with breastfeeding period.

Factors associated with a “less than expected improvement in CD4 cell counts” at the six -month visits were age, WHO clinical stage and CD4 cell counts at first ANC visit. Women aged 20–24 years (aOR = 2.27, 95% CI: 1.17; 4.40), those severely immunocompromised in WHO clinical stage IV (aOR = 9.59; 95% CI: 2.94;31.27) and with a CD4 cell count ≥ 200 (aOR = 1.92, 95% CI: 0.97;3.83) had higher odds of a “less than expected improvement in the CD4 cell count” while on ART compared to younger women (aged < 20 years), those in WHO clinical stage I-II and with a CD4 cell count < 200, respectively. Likewise, the Multivariate Imputation by Chained Equations sensitivity analyses did not find substantial differences, suggesting no major impact of missing data on the results (Tables 5b-Supplementary Information) Table 5.

**Table 5:** Factors associated with less than expected improvement in the CD4 + cell count over time by advanced HIV disease status

Variable			Unadjusted			Adjusted		
			OR	95%CI	*P*-value	OR	95% CI	*P*-value
Age group (years)		< 20	1		0.012	1		0.018
		20–24	2.24	1.16; 4.34		2.27	1.17; 4.40	
		25–34	1.31	0.7; 2.48		1.34	0.71; 2.55	
		> 35	1.59	0.77; 3.30		1.56	0.74; 3.28	
Gestational age at first ANC visit			1.00	0.97; 1.03	0.957	1.17	0.8; 1.71	0.954
WHO Clinical Stage		I	1		< 0.001	1		0.002
		II	0.66	0.31; 1.40		0.73	0.34; 1.56	
		III	0.80	0.36; 1.79		0.87	0.39; 1.98	
		IV	8.33	2.61; 26.56		9.59	2.94; 31.27	
CD4 count (cells/mm^3^)		< 200	1		0.056	1		0.045
		≥ 200	1.73	0.89; 3.83		1.92	0.97; 3.83	
ART start	Before first ANC visit		1		0.606	1		0.417
	After first ANC visit		1.13	0.78; 1.62		1.34	0.79; 2.27	

*P* value (LR-test). Number of observations: 1298 (unadjusted and adjusted ORs were based only on observations with complete information on all variables). *ART* Antiretroviral therapy, *ANC* Antenatal care, *WHO* Word health organization

### Maternal and neonatal outcomes

Information on maternal and neonatal outcomes was available for 1534 out of 2458 study participants (62%) (Table 6). Median follow-up was 1150 (IQR: 634.5–1527) days in women with AHD and 1046 (IQR: 562–1494) in women without AHD. Most women were on ART at the time of delivery [188 (96.4%) of those with AHD and 1298 (97.4%) of those without it] and one month after delivery [195 (99.0%) of those with AHD and 1331 (99.8%) of those without it]. No significant differences were detected between women with and without AHD regarding the type of delivery, frequency of prematurity, low birth weight, neonate HIV status and birth defects. Fifteen maternal deaths were reported during the 36 months study follow-up. No significant differences were detected between women with and without AHD regarding maternal mortality in women with AHD [1.1% (4/349)] *versus* [0.5% (11/2109)] (OR: 2.1; 95%CI 0.70–6.98; *p* = 0.309). The majority of these deaths occurred within six months after the first ANC clinic visit (40%) and among women who were not receiving ART at enrolment (53.3%). According to the Kaplan–Meier survival curves up until 36 months after the first ANC visit, all pregnant women with and without AHD appeared to have similar probabilities for death (*p* = 0.750).

**Table 6:** Maternal and perinatal outcomes among women with and without advanced HIV disease

Variable			Advanced HIV disease		*P*-value
			Yes *N* = 197	No *N* = 1337	
At the delivery					
Mother on ART			188 (96.4)	1298 (97.4)	0.549
Gestational age (weeks): median (IQR)			38 (36–40)	39 (37–40)	0.052
Type of delivery		Vaginal	187 (95.9)	1284 (96.4)	0.887
		Caesarean	8 (4.1)	48 (3.6)	
Preterm birth			20 (11.9)	99 (8.4)	0.187
Birthweight (grams): median (IQR)			3000 (2800–3275)	3000 (2800–3400)	0.079
Low birthweight (< 2500 g)			24 (12.4)	137 (10.3)	0.457
One month within delivery					
Mother on ART			195 (99.0)	1331 (99.6)	0.617
Breastfeeding			191 (97.0)	1309 (97.9)	0.276
Infant on cotrimoxazole prophylaxis			172 (88.7)	1210 (90.8)	0.401
Neonate HIV infected			5 (3.9)	14 (1.6)	0.078
At the end of follow up period^a^					
Variable			*N* = 349	*N* = 2109	
Deaths			4 (1.1)	11 (0.5)	0.309
Period of death after ANC enrolment	0–5 months		2 (50.0)	4 (36.4)	0.821
	6–11 months		1 (25.0)	1 (9.1)	
	12–17 months		0	1 (9.1)	
	18–23 months		0	1 (9.1)	
	≥ 24 months		1 (25.0)	4 (36.4)	

*P*-value for categorical variables is from Fisher exact test, and for continuous variables is from Mann–Whitney U test. Prematurity: child born at less than 37 weeks' gestation. *IQR* Interquartile range, *ART* Antiretroviral therapy, *ANC* Antenatal care. ^a^Using HDSS data base

## Discussion  

To our knowledge, this study is the first one describing the burden, clinical presentation and risk factors for AHD in Mozambican pregnant women. Despite the roll out of ART in the last two decades in the country, we observed that at first ANC visit over 14% of HIV-infected pregnant women presented with AHD and about 2% of them were severely immunosuppressed. In addition, there were no significant changes in the annual frequency of pregnant women with AHD starting ANC between 2015 and 2020. This unacceptable and constant high rate of AHD among pregnant women has important clinical and public health implications, including the strengthening of ongoing HIV-control efforts specially focused in women of childbearing age. The proportion of women with AHD in this cohort was lower than the 37% estimated  in the general population in Mozambique, although this figure is from 2014 [8], and similar to reports in pregnant women from neighboring South Africa (12%) and Tanzania (12-14%) [13, 38, 39]. The results of this study showed that a high proportion (76.2%) of pregnant women with AHD had been on ART for a long period (nearly 2 years), suggesting suboptimal case management regarding detection of low adherence and/or treatment failure before the current pregnancy. In fact, despite reports of high prevalence of HIV drug resistance in pregnant women in Mozambique [40], only a small proportion (3%) of women with AHD were receiving the second line ART regimen (AZT/3TC/LPV/r or ABC), while 10.9% were on the less effective AZT/3TC/NVP regimen [41]. Over 14% of the women who presented with AHD at the first ANC visit were not receiving ART indicating timeworn HIV infection before the current pregnancy and a missed opportunity of being enrolled on ART before HIV disease progression. In the current study, tuberculosis was the only opportunistic infection identified in pregnant women with AHD; however, this does not exclude the existence of other opportunistic infections [12]. It could be hypothesized that since bacterial infections often present with acute symptoms, women with these infections may have sought medical assistance before attending the ANC clinic. In addition, the provision of cotrimoxazole prophylaxis may have been effective in reducing the risk of bacterial infections [42–44]. Additionally, as in other low-income settings, limited access to diagnostic facilities may explain the low detection of opportunistic infections, supporting the need for availability of accurate diagnostic tests in these settings [45, 46]. There was an increasing trend of the median CD4+ cell counts in women without AHD during the 36 months follow-up, however, this remained constantly below 200 cells/mm³ in women with AHD at first ANC visit. Furthermore, fewer women with AHD had an increase in CD4 cell counts over time during post-partum suggesting that a possible improvement in ART adherence during pregnancy did not continue after delivery. Thus, approaches to increase ART adherence after delivery will be useful to prevent mother-to-child HIV transmission through breastfeeding while improving women’s health. The CD4 drop in pregnancy (about 50 cells) is not a real reduction in CD4 cells but the same amount in a larger amount of blood due to hemodilution. The drop is only temporary, which is more in the first trimester of pregnancy and in primigravidae [19]. In this study, we calculated variation in CD4 cell counts from the first ANC visit, on what he median (IQR) gestational age at ANC enrolment was 21 [15–25] weeks, therefore, after the first trimester of pregnancy. In 12.5% of the HIV-infected women with AHD at the six month visit and in 14.2% of the HIV-infected women without AHD at the 12 month visit after the first ANC, a “less than expected improvement in CD4+ cell counts” was observed, with a reduction in the CD4+ cell count of 274.5 cells/mm³ and of 117.7 cells/mm³, respectively. This finding indicates that AHD would not be detected in many women with borderline CD4+ cell counts (CD4+ cell count <200 cells/mm³ for AHD definition) in the absence of repeated measurements of CD4+ cell counts.  Currently, viral load measurement is recommended to monitor the ART response, however, in Mozambique, only 61% of people living with HIV on ART had had at least one viral load test [47]. Therefore, efforts are needed to improve access to viral load testing facilities. In addition, given the high burden of infectious diseases in many low-income settings these results support the recommendation for continued CTXp for all women of childbearing age regardless of the CD4+ cell count [42–44]. Our findings are consistent with previous studies showing that older age and ART regimen (AZT/3TC/NVP and AZT/3TC/LPV/r or ABC) are risk factors for presenting AHD [12, 35, 36], and also with reports showing that older age and being on WHO Clinical Stage IV  were risk factors for “less than expected improvement in CD4 cell counts” [29, 48]. Assessment of these factors may help to identify HIV-infected pregnant women with AHD and thus, prioritize case management and prevention of disease progression. The fact that women with CD4<200 cell counts at the first ANC visit had a lower risk of “less than expected improvement in CD4 cell counts” six months later could be explained by the fact that women identified with AHD may be more closely monitored compared with those without it, which might improve adherence to ART. In any case, the confounding effect of some other variable not included in the logistic regression analysis and which could explain our discordant results cannot be ruled out. We found no differences in the frequency of adverse maternal and perinatal outcomes between women with and without AHD, which might be explained by the overall high ART adherence rate at the end of pregnancy. At the time of delivery and one month postpartum, the majority of women were retained on ART. These results support the benefits of ART on preventing adverse pregnancy outcomes among women with AHD disease [49, 50]. While some limitations of the study have been discussed previously, other methodological limitations should be mentioned.  The descriptive study design did not allow us to make direct assessments of the impact of different interventions implemented during study period. Other limitation of this study is due to the fact that since March 2019, repeated CD4+ cell counts measurements were only performed in women with suspicion of AHD, and therefore, in the last year of the study, the proportion of women with a “less than expected improvement in CD4+ cell counts” after the first ANC visit may have been overestimated. In addition, information on participant’s vital status (mortality) were retrieve from HDSS data base with poor information on the mortality causes. Finally, due to the retrospective design of part of the study, there were missing values in some important study variables, such as viral load, limiting the identification of women who may have had immunological discordant response to ART and validity of all the analyses. Nevertheless, the Multivariate Imputation by Chained Equations sensitivity analyses did not find substantial differences, suggesting no major impact of missing data and consistency of the results.

## Conclusion

After more than two decades of ART roll out, 14% of HIV-infected pregnant women presented with AHD disease at first ANC visit in this area of southern Mozambique. In order to achieve progress towards the UNAIDS 95/95/95 goals for 2025 in HIV-infected pregnant women, prompt HIV diagnosis in women of childbearing age, effective linkage to HIV care with an optimal ART regimen and close monitoring after ART initiation, are fundamental strategies that must be urgently implemented to improve maternal and child survival. 

## Supplementary Information

**Additional file 1: Table 2-b**. Independent risk factors for advanced HIV disease (according to multiple imputation analyses).

**Additional file 2: Table 5-b**. Factors associated with less than expected improvement in the CD4+ cell count over time by advanced HIV disease status (according to multiple imputation analyses).

## Data Availability

The datasets used and/or analyzed during the current study are available from the corresponding author on reasonable request.

## Abbreviations

ABC: Abacavir
ANC: Antenatal care
AHD: Advanced HIV disease
ART: Antiretroviral therapy
ARV: Antiretroviral
AZT: Zidovudine
CISM: *Centro de Investigação em Saúde de Manhiça*
CTXp: Cotrimoxazole prophylaxis
DHSS: Demographic and Health Surveillance System
DTG: Dolutegravir
EFV: Efavirenze
IeDEA-SA: International Epidemiological Databases to Evaluate AIDS in Southern Africa
LPV/r: Lopinavir-ritonavir
NID: Numeric identifier
NVP: Nevirapine
PMTCT: Prevention of mother to child transmission
TDF: Tenofovir
3TC: Lamivudine

## References

[1] UNAIDS. Country factsheets [Internet]. Available from: https://www.unaids.org/es/regionscountries/countries/mozambique. Accessed 20 Nov 2021.

[2] Inquérito de Indicadores de Imunização, Malária e HIV/SIDA em Moçambique (IMASIDA) 2015: Relatório de Indicadores Básicos de HIV. Instituto Nacional de Saúde, Instituto Nacional de Estatística de Moçambique. 2017. Available from: https://dhsprogram.com/pubs/pdf/AIS12/AIS12.pdf. Accessed 23 Nov 2021.

[3] González R, Munguambe K, Aponte J, Bavo C, Nhalungo D, Macete E (2012). High HIV prevalence in a southern semi-rural area of Mozambique: a community-based survey. HIV Med..

[4] MISAU. 2020 HIV/AIDS Report. Mozambique Ministry of Health. 2021. Available from: https://www.misau.gov.mz/index.php/relatorios-anuais2021. Accessed 20 Nov 2021.

[5] Acceleration Plan for the Response to HIV/AIDS. Mozambique Ministry of Health. 2013. Available from: http://www.misau.gov.mz/index.php/planos-estrategicos-do-hiv. Accessed 20 Nov 2021.

[6] WHO. Consolidated Guidelines on The Use of Antiretroviral Drugs for Treating And Preventing HIV infection [Internet]. WHO. 2013 [cited 2010 Jul 20]. Available from: http://apps.who.int/iris/bitstream/10665/85321/1/9789241505727_%0Aeng.pdf?ua=1. Accessed 22 Nov 2021.

[7] World Health Organization. HIV/AIDS Programme. Programmatic Update. Use of Antiretroviral Drugs for Treating Pregnant Women and Preventing HIV Infections in Infants. Available from: https://apps.who.int/iris/bitstream/handle/10665/70892/WHO_HIV_2012.6_por.pdf?sequence=1&isAllowed=y. 2021. Accessed 28 Dec 2021.

[8] Auld AF, Shiraishi RW, Couto A, Mbofana F, Colborn K, Alfredo C (2016). A Decade of Antiretroviral Therapy Scale-up in Mozambique. JAIDS J Acquir Immune Defic Syndr..

[9] UNAIDS. Understanding fast-track accelerating action to end the AIDS epidemic by 2030. 2015. Available from: https://www.unaids.org/sites/default/files/media_ asset/ JC2686_WAD2014report_en.pdf. Accessed 28 Dec 2021.

[10] Ford N, Shubber Z, Meintjes G, Grinsztejn B, Eholie S, Mills EJ (2015). Causes of hospital admission among people living with HIV worldwide: a systematic review and meta-analysis. lancet HIV..

[11] Low A, Gavriilidis G,  Larke N, -Lajoie M-R B,  Drouin O, J Stover (2016). Incidence of Opportunistic Infections and the Impact of Antiretroviral Therapy Among HIV-Infected Adults in Low- and Middle-Income Countries: A Systematic Review and Meta-analysis. Clin Infect Dis..

[12] World Health Organization. guidelines for managing advanced hiv disease and rapid initiation of antiretroviral therapy, July 2017 WHO [Internet]. Available from: http://apps.who.int/iris/bitstream/handle/10665/255884/9789241550062-eng.pdf;jsessionid=11829ECD3684DE92B900B713F4C6255F?sequence=1. Accessed 20 Nov 2021. 29341560

[13] Auld AF, Shiraishi RW, Oboho I, Ross C, Bateganya M, Pelletier V (2017). Trends in Prevalence of Advanced HIV Disease at Antiretroviral Therapy Enrollment — 10 Countries, 2004–2015. MMWR Morb Mortal Wkly Rep..

[14] Cates JE, Westreich D, Edmonds A, Wright RL, Minkoff H, Colie C (2015). The Effects of Viral Load Burden on Pregnancy Loss among HIV-Infected Women in the United States. Infect Dis Obstet Gynecol..

[15] Chilaka VN, Konje JC (2021). HIV in pregnancy - An update. Eur J Obstet Gynecol Reprod Biol..

[16] Calvert C, Ronsmans C (2013). The contribution of HIV to pregnancy-related mortality: a systematic review and meta-analysis. AIDS..

[17] Calvert C, Ronsmans C (2015). Pregnancy and HIV disease progression: a systematic review and meta-analysis. Trop Med Int Heal..

[18] Ekouevi DK, Inwoley A, Tonwe-Gold B, Danel C, Becquet R, Viho I (2007). Variation of CD4 Count and Percentage during Pregnancy and after Delivery: Implications for HAART Initiation in Resource-Limited Settings. AIDS Res Hum Retroviruses..

[19] Byrns M, Elwood C, Capmas P, Kakkar F, Boucher M, Money D (2020). Variation of CD4 count in pregnant women living with HIV. Am J Obstet Gynecol..

[20] Wall KM, Rida W, Haddad LB, Kamali A, Karita E, Lakhi S (2017). Pregnancy and HIV Disease Progression in an Early Infection Cohort from Five African Countries. Epidemiology..

[21] Nhacolo A, Jamisse E, Augusto O, Matsena T, Hunguana A, Mandomando I, et al. Cohort profile update: Manhiça health and demographic surveillance system (HDSS) of the Manhiça health research centre (CISM). Int J Epidemiol. 2021 Jan 16; https://doi.or/10.1093/ije/dyaa218. 10.1093/ije/dyaa218 PMC8128467 33452521

[22] Bassat Q, Guinovart C, Sigaúque B, Aide P, Sacarlal J, Nhampossa T, et al. Malaria in rural Mozambique. Part II: Children admitted to hospital. Malar J. 2008;7. https://doi.or/10.1093/ije/dyaa218. 10.1186/1475-2875-7-37 PMC2275288 18302771

[23] Aide P, Aponte JJ, Renom M, Nhampossa T, Sacarlal J, Mandomando I, et al. Safety, immunogenicity and duration of protection of the RTS,S/ASO2<inf>D</inf> malaria vaccine: One year follow-up of a randomized controlled phase I/IIb trial. PLoS One. 2010;5(11). 10.1371/journal.pone.0013838. 10.1371/journal.pone.0013838 PMC2973956 21079803

[24] Lambdin BH, Micek MA, Koepsell TD, Hughes JP, Sherr K, Pfeiffer J (2012). An assessment of the accuracy and availability of data in electronic patient tracking systems for patients receiving HIV treatment in central Mozambique. BMC Health Serv Res..

[25] MISAU. Introdução de Normas Clinicas Atualizadas e dos Modelos Diferenciados de Serviços, para o seguimento do paciente HIV positivo – CIRCULAR 2. 2019. Available from: https://comitetarvmisau.co.mz/docs/orientacoes_nacionais/ Circular_Normas_Cli%CC%81nicas_08_03_19.pdf. Accessed 12 Jan 2022.

[26] MISAU. Tratamento Antiretroviral e Infecções Oportunistas do Adulto, Adolescente, Grávida e Criança. 2016. Available from: file:///C:/Users/tnhampossa/Downloads/ Tratamento%20Antiretroviral%20e%20Infec%C3%A7%C3%B5es%20Oportunistas%20no%20Adulto%20Adolescente%20Gr%C3%A1vida%20e%20Crian%C3%A7a%20-%20Gui%C3%A3o%20de%20Bolso%20(1).pdf. Accessed 14 May 2017.

[27] MISAU. Circular Normas Clínicas 08.03.19 - COMITÉ TARV [Internet]. Available from: omitetarvmisau.co.mz/docs/orientacoes_nacionais/Circular_Normas_Clínicas_08_03_19.pdf. Accessed 12 Jan 2022.

[28] Zaniewski E, Tymejczyk O, Kariminia A, Desmonde S, Leroy V, Ford N, et al. IeDEA-WHO Research-Policy Collaboration: contributing real-world evidence to HIV progress reporting and guideline development. J virus Erad. 2018 Nov 15;4(Suppl 2):9–15. Available from: https://www.ncbi.nlm.nih.gov/pmc/articles/PMC6248847/. Accessed 12 Jan 2022. 10.1016/S2055-6640(20)30348-4 PMC6248847 30515309

[29] Mocroft A, Phillips AN, Gatell J, Ledergerber B, Fisher M, Clumeck N (2007). Normalisation of CD4 counts in patients with HIV-1 infection and maximum virological suppression who are taking combination antiretroviral therapy: an observational cohort study. Lancet (London, England).

[30] Loutfy MR, Genebat M, Moore D, Raboud J, Chan K, Antoniou T, et al. A CD4+ Cell Count <200 Cells per Cubic Millimeter at 2 Years After Initiation of Combination Antiretroviral Therapy Is Associated With Increased Mortality in HIV-Infected Individuals With Viral Suppression. JAIDS J Acquir Immune Defic Syndr. 2010 Dec 1;55(4):451–9. 10.1097/qai.0b013e3181ec28ff. 10.1097/qai.0b013e3181ec28ff 21105259

[31] Ahmed T, Hossain M, Sanin KI (2012). Global Burden of Maternal and Child Undernutrition and Micronutrient Deficiencies. Ann Nutr Metab..

[32] Adam I, Ali AA. Anemia During Pregnancy. In: Nutritional Deficiency. InTech; 2016.

[33] Romano M, Cacciatore A, Giordano R, La Rosa B. Postpartum period: three distinct but continuous phases. J Prenat Med. 2010 Apr;4(2):22–5. Available from: http://www.ncbi.nlm.nih.gov/pubmed/22439056 PMC3279173 22439056

[34] Taha Z, Ali Hassan A, Wikkeling-Scott L, Papandreou D (2020). Factors Associated with Preterm Birth and Low Birth Weight in Abu Dhabi, the United Arab Emirates. Int J Environ Res Public Health..

[35] Jiang H, Liu J, Tan Z, Fu X, Xie Y, Lin K (2020). Prevalence of and factors associated with advanced HIV disease among newly diagnosed people living with HIV in Guangdong Province. China J Int AIDS Soc.

[36] Lebelonyane R, Mills LA, Mogorosi C, Ussery F, Marukutira T, Theu J (2020). Advanced HIV disease in the Botswana combination prevention project: prevalence, risk factors, and outcomes. AIDS.

[37] Buuren S van, Groothuis-Oudshoorn K. mice. Multivariate Imputation by Chained Equations in R. J Stat Softw. 2011;45(3). 10.18637/jss.v045.i03.

[38] Malaba TR, Phillips T, Le Roux S, Brittain K, Zerbe A, Petro G (2017). Antiretroviral therapy use during pregnancy and adverse birth outcomes in South African women. Int J Epidemiol..

[39] Shayo GA, Moshiro C, Spiegelman D, Mugusi FM, Chalamilla G, Msamanga G (2014). Prevalence and risk factors for skin diseases among antiretroviral-naïve HIV-infected pregnant women in Dar es Salaam. Tanzania. Int J Dermatol..

[40] Rupérez M, Noguera-Julian M, González R, Maculuve S, Bellido R, Vala A (2018). HIV drug resistance patterns in pregnant women using next generation sequence in Mozambique Ceccherini-Silberstein F, editor. PLoS One..

[41] Cain LE, Phillips A, Lodi S, Sabin C, Bansi L, Justice A, et al. The effect of efavirenz versus nevirapine-containing regimens on immunologic, virologic and clinical outcomes in a prospective observational study. AIDS. 2012 Aug 24;26(13):1691–705. 10.1097/QAD.0b013e328354f497. 10.1097/QAD.0b013e328354f497 PMC3647467 22546987

[42] Suthar AB, Vitoria MA, Nagata JM, Anglaret X, Mbori-Ngacha D, Sued O, et al. Co-trimoxazole prophylaxis in adults, including pregnant women, with HIV: a systematic review and meta-analysis. Lancet HIV. 2015 Apr;2(4):e137-50. 10.1016/S2352-3018(15)00005-3. 10.1016/S2352-3018(15)00005-3 26424674

[43] Campbell JD, Moore D, Degerman R, Kaharuza F, Were W, Muramuzi E, et al. HIV-Infected Ugandan Adults Taking Antiretroviral Therapy With CD4 Counts >200 Cells/ L Who Discontinue Cotrimoxazole Prophylaxis Have Increased Risk of Malaria and Diarrhea. Clin Infect Dis. 2012 Apr 15;54(8):1204–11. 10.1093/cid/cis013. 10.1093/cid/cis013 22423133

[44] Polyak  CS, Yuhas K, Singa B, Khaemba M, Walson J, Richardson BA (2016). Cotrimoxazole Prophylaxis Discontinuation among Antiretroviral-Treated HIV-1-Infected Adults in Kenya: A Randomized Non-inferiority Trial.  Carr A, editor. PLOS Med.

[45] Johansen ØH, Abdissa A, Zangenberg M, Mekonnen Z, Eshetu B, Bjørang O, et al. Performance and operational feasibility of two diagnostic tests for cryptosporidiosis in children (CRYPTO-POC): a clinical, prospective, diagnostic accuracy study. Lancet Infect Dis. 2021 May;21(5):722–30. 10.1016/S1473-3099(20)30556-9. 10.1016/S1473-3099(20)30556-9 PMC8064915 33278916

[46] McNerney R. Diagnostics for Developing Countries. Diagnostics (Basel, Switzerland). 2015 May 19;5(2):200–9. 10.3390/diagnostics5020200. 10.3390/diagnostics5020200 PMC4665590 26854149

[47] MISAU. 2021 HIV/AIDS Report. 2022. Available from: https://www.misau.gov.mz/index.php/relatorios-anuais

[48] Loutfy MR, Genebat M, Moore D, Raboud J, Chan K, Antoniou T, et al. A CD4+ cell count <200 cells per cubic millimeter at 2 years after initiation of combination antiretroviral therapy is associated with increased mortality in HIV-infected individuals with viral suppression. J Acquir Immune Defic Syndr. 2010 Dec;55(4):451–9. 10.1097/qai.0b013e3181ec28ff. 10.1097/qai.0b013e3181ec28ff 21105259

[49] Fowler MG, Qin M, Fiscus SA, Currier JS, Flynn PM, Chipato T (2016). Benefits and Risks of Antiretroviral Therapy for Perinatal HIV Prevention. N Engl J Med..

[50] Uthman OA, Nachega JB, Anderson J, Kanters S, Mills EJ, Renaud F, et al. Timing of initiation of antiretroviral therapy and adverse pregnancy outcomes: a systematic review and meta-analysis. Lancet HIV. 2017 Jan;4(1):e21-30. 10.1016/S2352-3018(16)30195-3. 10.1016/S2352-3018(16)30195-3 27864000

