# Supplementary material for: Burden, clinical presentation and risk factors of advanced HIV disease in pregnant Mozambican women
Source: BMC Pregnancy Childbirth. 2022 Oct 8;22:756. doi: 10.1186/s12884-022-05090-3 (PMC9548114; doi:10.1186/s12884-022-05090-3)
Supplement: Supplementary file 1 — Additional file 1: Table 2-b. Independent risk factors for advanced HIV disease (according to multiple imputation analyses). [file 12884_2022_5090_MOESM1_ESM.docx]

**Table 2-b.** Independent risk factors for advanced HIV disease (according to multiple imputation analyses)

| Variable | | | Unadjusted | | | Adjusted | | |
| --- | --- | --- | --- | --- | --- | --- | --- | --- |
|  |  |  | OR | 95%CI | P-value | OR | 95% CI | P-value |
| Age group | | <20 | 1 |  |  | 1 |  | 0.025 |
|  |  | 20-24 | 1.40 | 0.69-2.84 |  | 1.39 | 0.69; 2.83 |  |
|  |  | 25-34 | 1.90 | 1.30-3.62 |  | 1.86 | 0.97; 3.59 |  |
|  |  | >35 | 2.73 | 1.34-5.56 |  | 2.61 | 1.26; 5.41 |  |
| Gestational age at the first ANC visit | | | 0.98 | 0.95; 1.00 | 0.100 | 0.98 | 0.96; 1.01 | 0.130 |
| ART start | Before first ANC visit | | 1 |  | 0.633 | 1 |  | 0.282 |
|  | After first ANC visit | | 1.09 | 0.76; 1.55 |  | 1.23 | 0.85; 1.77 |  |
| ART Regime | TDF/3TC/EFV | | 1 |  | <0.001 | 1 |  | <0.001 |
|  | AZT/3TC/NVP | | 3.48 | 2.05-5.91 |  | 3.21 | 1.87; 5.53 |  |
|  | AZT/3TC/LPV/r or ABC | | 7.59 | 2.42; 23.81 |  | 6.93 | 2.18; 22.05 |  |
| Body mass index | | | 0.97 | 0.93; 1.01 | 0.131 | 0.96 | 0.92; 1.00 | 0.071 |
