# Supplementary material for: Burden, clinical presentation and risk factors of advanced HIV disease in pregnant Mozambican women
Source: BMC Pregnancy Childbirth. 2022 Oct 8;22:756. doi: 10.1186/s12884-022-05090-3 (PMC9548114; doi:10.1186/s12884-022-05090-3)
Supplement: Supplementary file 2 — Additional file 2: Table 5-b. Factors associated with less than expected improvement in the CD4+ cell count over time by advanced HIV disease status (according to multiple imputation analyses). [file 12884_2022_5090_MOESM2_ESM.docx]

**Table 5-b.** Factors associated with less than expected improvement in the CD4+ cell count over time by advanced HIV disease status (according to multiple imputation analyses)

| Variable | | | Unadjusted | | | Adjusted | | |
| --- | --- | --- | --- | --- | --- | --- | --- | --- |
|  |  |  | OR | 95%CI | P-value | OR | 95% CI | P-value |
| Age group (years) | | <20 | 1 |  | 0.012 | 1 |  | 0.009 |
|  |  | 20-24 | 2.41 | 1.29; 4.52 |  | 2.44 | 1.30; 4.59 |  |
|  |  | 25-34 | 1.50 | 0.82; 2.75 |  | 1.57 | 0.85; 2.89 |  |
|  |  | >35 | 1.98 | 1.00; 3.92 |  | 2.05 | 1.02; 4.10 |  |
| Gestational age at first ANC visit | | | 0.997 | 0.97; 1.02 | 0.806 | 0.997 | 0.97; 1.02 | 0.799 |
| WHO Clinical Stage | | I | 1 |  | <0.001 | 1 |  | 0.001 |
|  |  | II | 0.52 | 0.25; 1.09 |  | 0.56 | 0.26; 1.18 |  |
|  |  | III | 0.78 | 0.37; 1.66 |  | 0.83 | 0.39; 1.78 |  |
|  |  | IV | 8.19 | 2.57; 26.05 |  | 8.97 | 2.76; 29.16 |  |
| CD4 count (cells/mm³) | | <200 | 1 |  | 0.107 | 1 |  | 0.039 |
|  |  | ≥200 | 1.66 | 0.90; 3.06 |  | 1.85 | 0.99; 3.47 |  |
| ART start | Before first ANC visit | | 1 |  | 0.210 | 1 |  | 0.187 |
|  | After first ANC visit | | 1.24 | 0.89; 1.72 |  | 1.26 | 0.90; 1.78 |  |
